# Supplementary material for: Dynamics of pore formation during laser powder bed fusion additive manufacturing
Source: Nat Commun. 2019 Apr 30;10:1987. doi: 10.1038/s41467-019-10009-2 (PMC6491446; doi:10.1038/s41467-019-10009-2)
Supplement: Supplementary file 1 — Supplementary Information [file 41467_2019_10009_MOESM1_ESM.pdf]

Supplementary Information:

Dynamics of pore formation during laser powder bed fusion additive manufacturing

Martin et al.

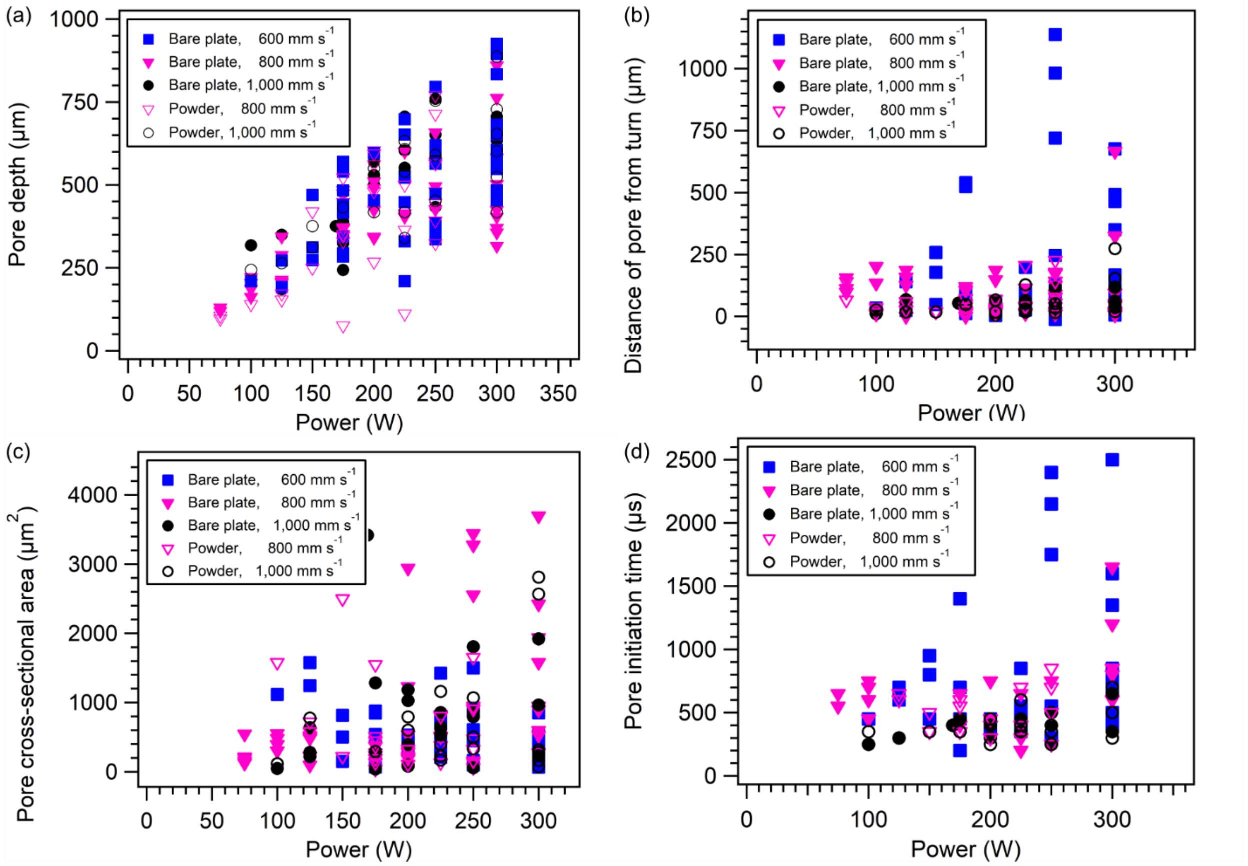

Supplementary Fig. 1. Full description of pore properties. Properties of pores formed during LPBF of Ti-6Al-4V in the laser turn point region as a function of laser power and steady state scan speed. All turn point condition scans were performed at full laser power. (a) Depth of pore relative to the substrate surface. (b) Distance of pore relative to the turn point. (c) Cross-sectional area of pore. (d) Pore initiation time (τ_p) after the laser completed the turn point.

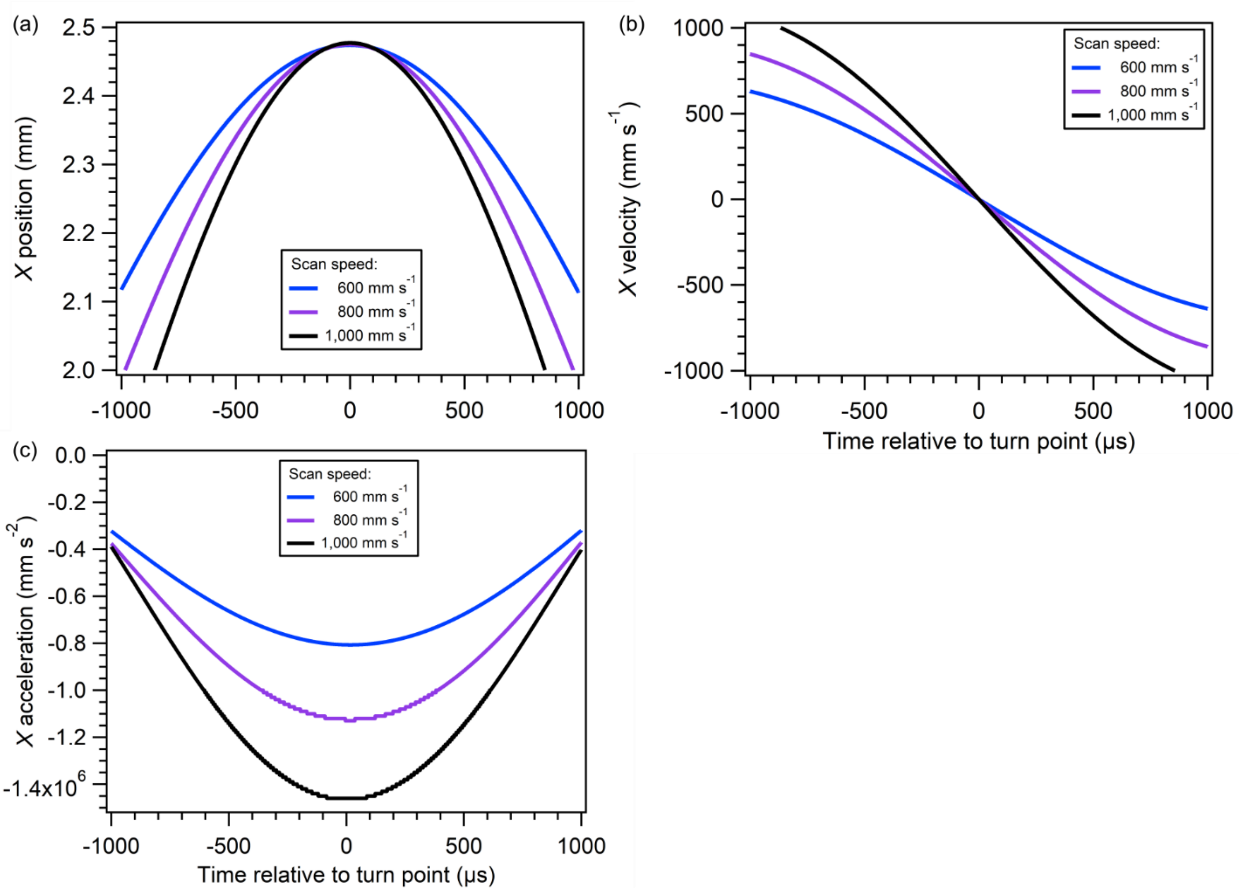

Supplementary Fig. 2. Mirror scanning properties during turn point. Scanning properties of the *X*-direction mirror during the turn point under various programmed steady state scan speeds. (a) Position, (b) velocity, and (c) acceleration of the *X*-direction mirror as a function of time relative to the turn point.

Supplementary Note 1: ALE3D multi-physics model details

Anisimov proposed a model where the recoil pressure, P, depends exponentially on temperature, $P(T) = 0.54P_\mathrm{a}\mathrm{e}^{-\frac{\lambda}{K_B}(\frac{1}{T}-\frac{1}{T_\mathrm{b}})}$, where P_a is the ambient pressure (1 Bar), λ is the evaporation energy per particle (4.3 eV per atom), K_B=8.617 x 10^{-5} eV K^{-1} is the Boltzmann constant, T is the surface temperature and T_b= is the boiling temperature of 316L stainless steel (3,086 K). According to Anisimov's model, approximately 18% of the metal vapor condenses back to the surface due to large angle scattering collisions in the vicinity of the liquid and hence reduces the evaporative cooling effect. The net material evaporation flux is $J_\mathrm{v} = \frac{0.82AP(T)}{\sqrt{2}\,\pi MRT}$ and is consistent with the recoil pressure, $P(T)$, derivation. A is a sticking coefficient, which is close to unity for metals, M is the molar mass, R the gas constant and T the surface temperature.

"Supplementary Video 1.avi" video was compiled in AVI format with JPEG compression at 30 simulation frames per second of video using ImageJ (version 1.50). The video shows the full sequence of simulation presented in Fig. 5 of the main manuscript (200 W constant laser power and 1,500 mm s^{-1} steady state scan speed).

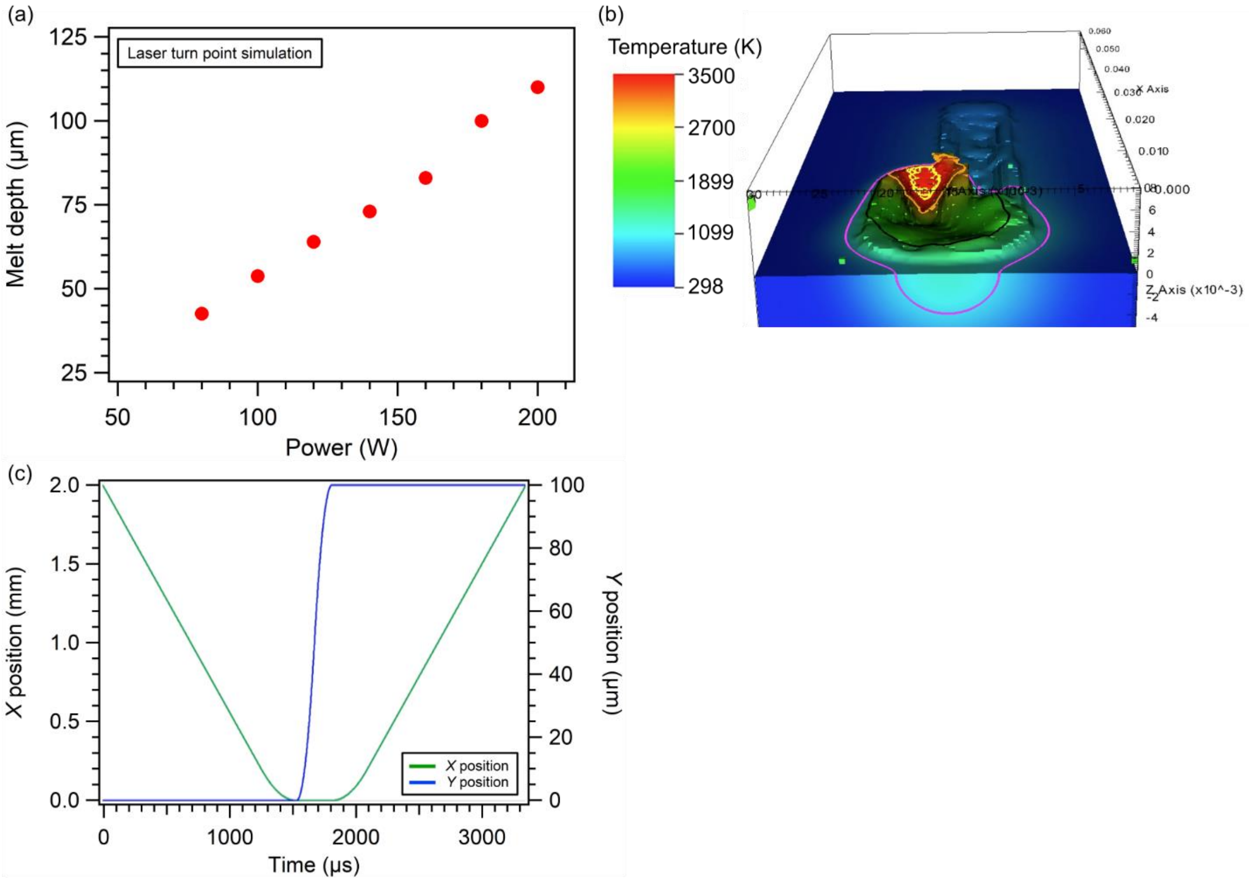

Supplementary Fig. 3. Simulations of turn point dynamics in SS316L. (a) Melt depth versus laser power at the laser turn point, showing a linear trend. (b) 3D view of a bare plate simulation in SS316L using ALE3D, showing the melt pool after the laser has scanned passed the turn point. The black contour line on the surface represents the melt pool boundary (1,700 K). The purple contour line indicates a material temperature of 1,000 K. Dimensions are all in centimeters. (c) *X* and *Y* position of the scanning mirrors used in the simulation as a function of time. Note the different scales of the Y-axes. The steady state speed was 1,500 mm s^{-1}.

Supplementary Note 2: In-process videos of vapor depression during constant power and mitigation strategy cases

"Supplementary Video 2.avi" video was compiled in AVI format with JPEG compression using ImageJ (version 1.50). X-ray images were collected using a camera exposure time of 25 µs per frame at 20 kHz and the AVI was compiled at 15 experimental frames per second of video.

The top and bottom panels of the video show the 100 W constant power and mitigation scan strategies respectively. Scans were performed at 1,000 mm s^{-1} programmed steady state scan speed with no powder on the surface.

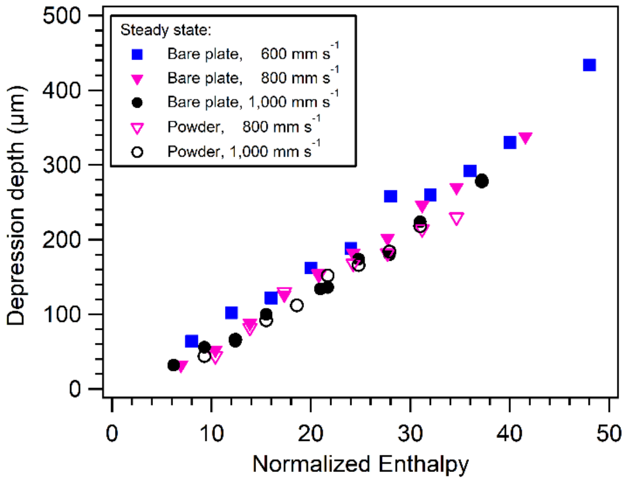

Supplementary Fig. 4. Normalized enthalpy under steady state conditions. Vapor depression depth during LPBF of Ti-6Al-4V as a function of normalized enthalpy.

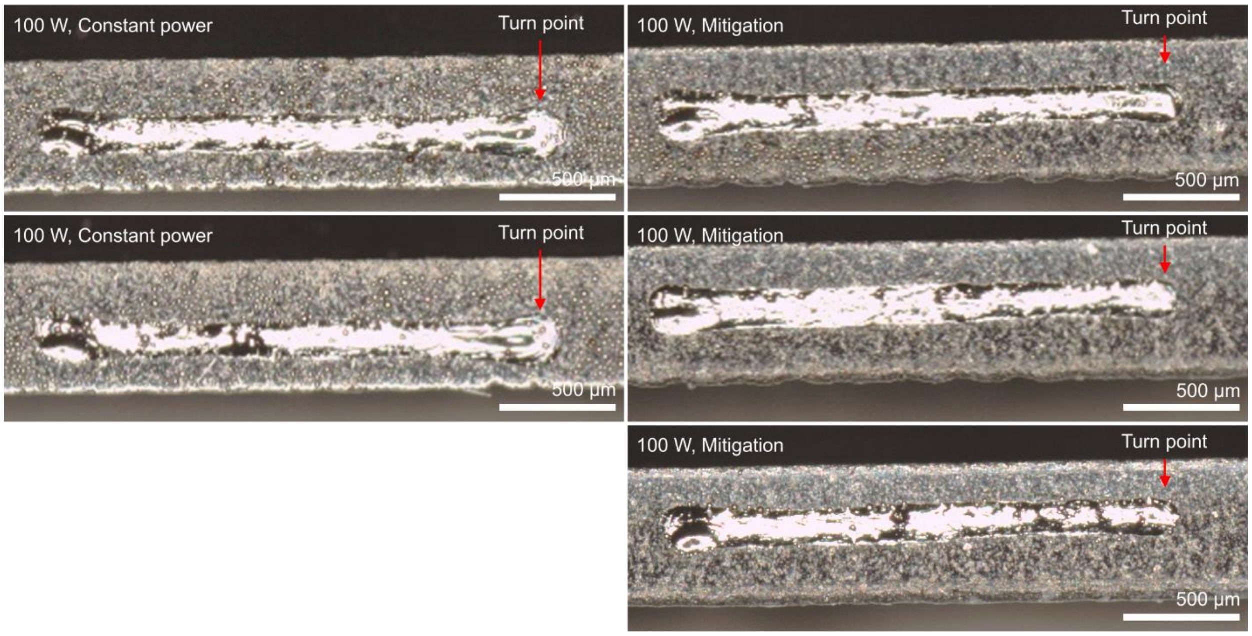

Supplementary Fig. 5. Optical images of tracks produced using constant power and mitigation strategy. Further examples of the quality of LPBF tracks produced in Ti-6Al-4V using the 100 W constant power and mitigated scan strategies. Tracks were produced using a 60 ± 20 μm thick layer of Ti-6Al-4V powder on Ti-6Al-4V plate.
